# Anterior Capsule Opening Contraction and Late Intraocular Lens Dislocation after Cataract Surgery in Patients with Weak or Partially Absent Zonular Support

**DOI:** 10.3390/medicina57010035

**Published:** 2021-01-03

**Authors:** Juris Vanags, Renārs Erts, Guna Laganovska

**Affiliations:** 1 Clinic of Ophthalmology, Pauls Stradins Clinical University Hospital, LV-1002 Riga, Latvia; guna.laganovska@rsu.lv; 2 Department of Ophthalmology, Riga Stradins University, LV-1007 Riga, Latvia; 3 Faculty of Medicine, University of Latvia, LV-1079 Riga, Latvia; renars.erts@lu.lv

**Keywords:** weak zonules, anterior capsule opening reduction, late intraocular lens dislocation, capsular tension ring

## Abstract

*Background and Objectives*: To evaluate anterior capsule opening (ACO) contraction and late intraocular lens (IOL) dislocation after cataract surgery in patients with weak or partially absent zonular support and assess methods of reducing these complications. *Materials and Methods*: For this prospective study, we enlisted cataract surgery patients in our hospital with preoperative diagnoses of weak zonules. All patients received phacoemulsification surgery with implantation of a hydrophobic acrylic IOL and capsular tension ring (CTR). ACO reductions were measured for six months after enrolment. Data on late IOL dislocations were collected five years after enrolment of the last patient. *Results*: Fifty-three patients were enrolled from 2011 to 2015. Over the six-month active follow-up period, ACO area reduction was 23% in patients receiving CTRs of 11 mm diameter and 8% for patients with CTRs of 12 mm, with an overall mean of 15% reduction. Five years after the last patient was enrolled, seven patients (13%) had experienced late IOL-CTR-capsular bag dislocation. For these patients, the mean ACO reduction in the first six months of follow-up was 33%, including for those who had received neodymium-doped yttrium aluminum garnet (Nd: YAG) anterior capsulotomies. *Conclusion*: Use of hydrophobic acrylic lenses and CTR reduces ACO contraction, with rates comparable to those after cataract surgery without ocular comorbidity. Our patients experienced a relatively high rate of late IOL-CTR-capsular bag dislocation. However, dislocated complexes were easily repositioned and few patients required IOL exchange. Frequent visits are warranted to promptly detect late complications of cataract surgery in patients with weak zonular support.

## 1. Introduction

Late intraocular lens (IOL) dislocation is a long-term, severe complication of non-complicated cataract phacoemulsification surgery involving continuous curvilinear capsulorhexis and in the bag IOL implantation [1,2,3,4]. Incidence of late IOL dislocation has increased in recent decades, reaching 0.3–1.2% from all cataract patients during some point of time after surgery [5,6,7,8]. The reported mean time of late IOL dislocation varies from 5 to 12.5 years after the initial cataract surgery [1,2,3,4,6,9]. Individual case reports from complicated surgeries have reported earlier occurrence, ranging from 2.5 to 8 years [10,11].

As defined by Davison, anterior capsule opening contraction is part of capsule contraction syndrome [12]. The syndrome is characterized by myofibroblastic transformation of lens epithelial cells (LEC), collagen synthesis, and contraction of the anterior capsule after cataract surgery. Myofibroblastic transformation of LEC is induced by increased amounts of cytokines (interleukin (IL)-1, IL-6, transforming growth factor (TGF) beta, and fibroblast growth factor) [13]. Among the multiple factors known to aggravate the condition are weak or absent zonular support to the lens capsule. More recently, elevated TGF-beta 2 and monocyte chemoattractant protein-1 levels have been defined as major causes of marked capsule contraction in the presence of weak zonules [14,15,16].

Weak or partially absent zonular support manifests in a variety of diseases, including pseudoexfoliation (PEX) syndrome [17,18], traumatic zonulolysis [19,20,21], multiple congenital abnormalities (Marfan syndrome, simple ectopia lentis, ectopia lentis et pupillae, Weil-Marchesani syndrome) [19], pathologic myopia and increased axial length [22], and retinitis pigmentosa [23,24]. These conditions are considered high-risk indicators for increased rates of late IOL dislocation related to progressive zonular dehiscence.

Numerous surgical approaches to treat the conditions have been developed, in case cataract surgery for these patients is necessary, which includes phacoemulsification with or without capsular tension ring implantation [22,25,26,27,28], different capsular bag and IOL fixation (sutured, glued) [11,29], and pars plana lentectomy combined with different types of IOL fixation [27,30]. In this prospective study we evaluated anterior capsule opening (ACO) contraction and late intraocular lens (IOL) dislocation and assessed the effectiveness of methods to reduce anterior capsule contraction after cataract surgery for patients with weak or absent zonules.

## 2. Materials and Methods

We enrolled 53 patients in our hospital from 2011 to 2015 and followed them for a minimum of five years after cataract surgery. Details of inclusion and exclusion criteria, examination and surgery techniques, and data on the first 30 patients have been previously published [31]. Eligible patients had preoperative signs of zonular weakness, specifically, phacodonesis, iridodonesis, or a lens margin visible in the pupillary area (grade 1–3 lens subluxation). The surgeries were performed with phacoemulsification and IOL implantation in the capsular bag. Small initial capsulorhexis (anterior capsule openings), if compared to other published data [32,33,34,35], were created due to the weak zonules.

Before starting the phacoemulsification, iris or capsular hooks along with a basic (11 or 12 mm) or Cionni (12 mm) capsular tension ring (CTR) were implanted to secure the capsular bag and support the anterior segment. Anterior capsule polishing to remove LEC was not performed in order to avoid additional stress to the zonules. To best fit patients’ individual conditions, we used five different IOL models from three manufacturers: the AcrySof SN60AT, MN60MA, and SN60WF (Alcon Surgical, Inc., Fort Worth, TX, USA), the Tecnis ZCB00 (Johnson & Johnson Vision, Santa Ana, CA, USA), and the 877FABY (Medicontur Medical Engineering Ltd., Zsámbék, Hungary). 

Anterior capsular contraction was observed at 1 day; 1 week; and 1, 3, and 6 months after the surgery. To measure the amount of contraction, we used a Carl Zeiss FF450^plus^ fundus camera with Visupac 4.3 software (Carl Zeiss Meditec AG, Jena, Germany) for photo-documentation of the anterior segment and calculation of anterior capsule opening, using an IOL diameter of 6 mm as reference. After the six-month examination, patients were instructed to return to our surgeon (J.V.) for cases of deteriorating visual acuity or other complaints, whether self-diagnosed or diagnosed by their local ophthalmologist. Five years after the last patient was enrolled in the initial study (the extended follow-up period), a local database was checked for possible ophthalmic events for those who had not returned for visits. These patients were contacted by telephone to clarify their current status or to invite them for ophthalmic examination. The size of the IOL’s optic area was calculated using the formula $S=\pi r^{2}$ (S = 3.14 × 3 × 3 = 28.26 mm^2^). The following equation was used to calculate the anterior capsular opening:

$$X=\frac{28.26\;\times \;B}{A},$$

where A is the IOL’s optic size, and B is the size of the capsulorrhexis, and both were obtained using measurements using the Fundus Camera’s Visupac software, and x is the real size of the capsulorrhexis.

The study protocol followed the tenets of the Declaration of Helsinki, and was approved by the Ethics Committee of the P. Stradins Clinical University Hospital (ID code: 080110-7L, 10 January, 2010). Written informed consent was obtained from patients during the initial study. 

### Statistical analysis

Patients’ demographic data and changes in their eyes are presented as means (M) and standard deviations (SD). Differences between groups were assessed using *t* tests for continuous variables and Pearson chi-square tests for discrete variables. Categorical variables were reported as count (percentage). Paired and unpaired *t* tests were applied when appropriate. Three groups were compared with ANOVA. All significance tests were 2-tailed, and *p* < 0.05 was used to indicate statistically significant differences. Statistical analyses were performed using R v.4.0.0 (R Foundation for Statistical Computing, Vienna, Austria).

## 3. Results

**Figure 1:**
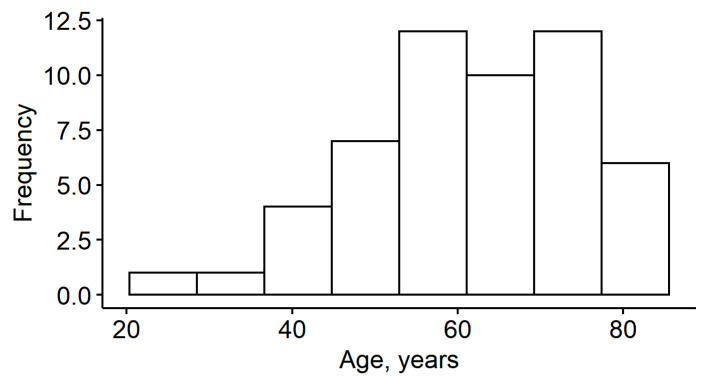
Histogram of patient age.

Fifty-three patients were enrolled in the study. Patients had a mean age of 62 years (SD = 14.0) and the majority (77%) were male (Table 1; Figure 1). Slightly over one-half (58%) of cataracts were in the left eye; 43% of the patients had glaucoma. Considering other risk factors, 11% of patients had myopia, 49% had a history of trauma, 6% had been diagnosed with Marfan syndrome, and 47% had PEX syndrome.

**Figure 2:**
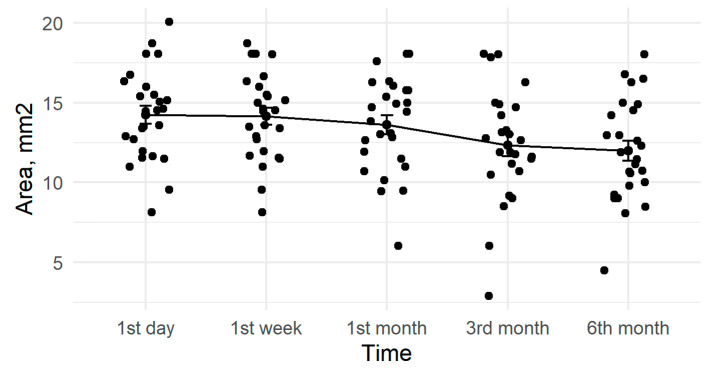
Amount of anterior capsule opening reduction during active follow-up.

**Figure 3:**
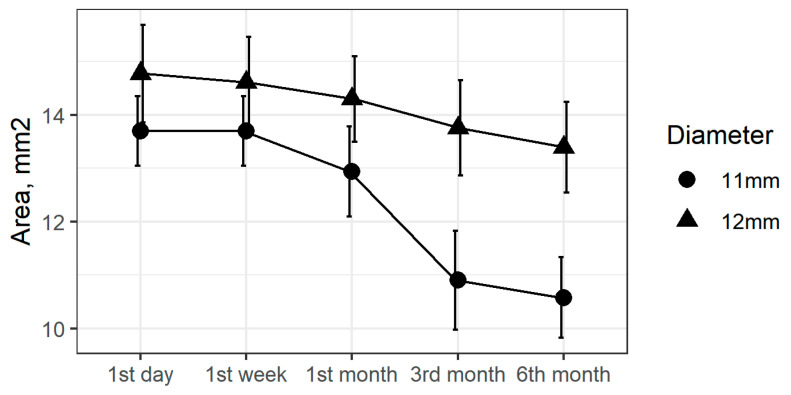
ACO area changes by capsular tension ring (CTR) diameter during active follow-up.

After six months, anterior capsule openings had reduced by an average of 15% (Figure 2). The six-month change in ACO diameter was statistically significant (*p* < 0.001, Table 2). Results differed based on the diameter of the CTR, with a mean reduction of 23% for the 11 mm CTRs and 8% for the 12 mm CTRs (Figure 3). 

Seven patients were deceased by the end of the extended follow-up period and nine could not be located. The local database did not have records of other ophthalmic surgeries for either of these groups. The lack of other surgeries for the deceased patients was confirmed by relatives contacted by telephone. We were unable to find contacts for the additional nine patients and a national database does not exist. Therefore, their lack of surgeries in other hospitals, including for management of late IOL dislocation, cannot be confirmed. 

Twenty-one patients returned to their local ophthalmologist after the last study visit, of whom two (10%) had suffered late IOL dislocation and undergone surgery in other clinics. Sixteen patients returned to our surgeon with complications. Of these patients, five (31%) had late IOL-CTR-capsular bag dislocation. Overall, seven patients (13%) experienced late IOL-CTR-capsular bag dislocation requiring surgical attention (Table 3). All of these dislocations were within the capsular bag. Six patients with late dislocation had had a basic CTR implanted during the initial surgery while one had received the Cionni CTR. The average time of late IOL-CTR-capsular bag dislocation was 57 months after surgery (SD = 35.6 months).

**Figure 4:**
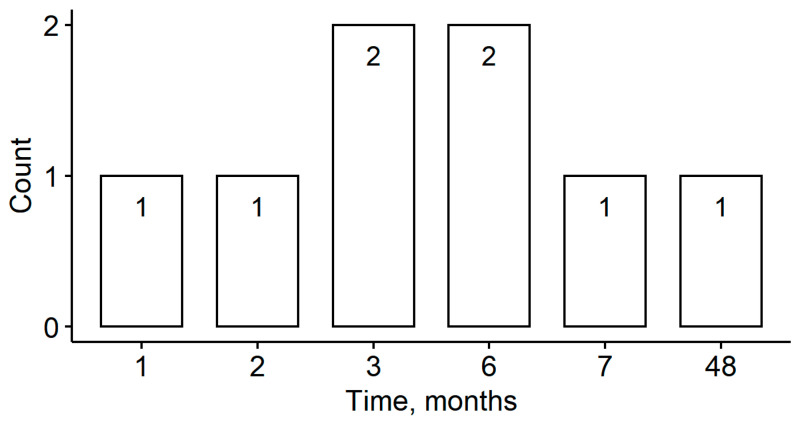
Timing of Nd: YAG anterior laser capsulotomies.

**Figure 5:**
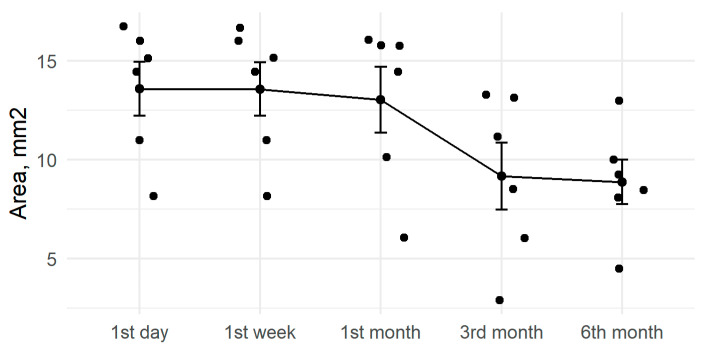
ACO reductions in patients with late intraocular lens (IOL)-CTR-capsular bag dislocations during active follow-up

**Figure 6:**
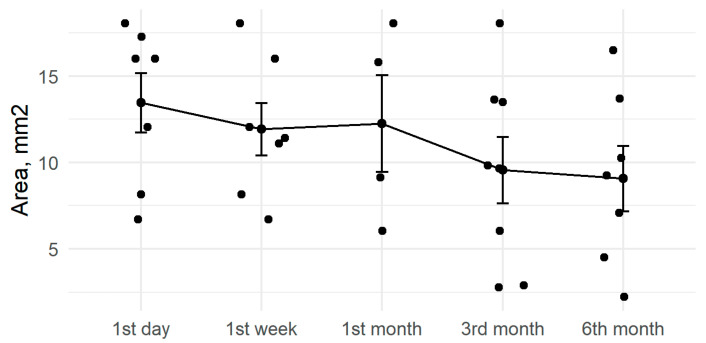
ACO reductions after neodymium-doped yttrium aluminum garnet (Nd: YAG) anterior capsulotomies.

Among the cohort of 53 patients, eight (15%) experienced significant ACO reduction during the follow-up period and received neodymium-doped yttrium aluminum garnet (Nd: YAG) anterior capsulotomy; the mean time of the procedure was ten months after the cataract surgery (Figure 4). Late IOL-CTR-capsular bag dislocation also occurred in two of these patients. The average ACO reduction over the first six months of follow-up was 33% for both late IOL-CTR-capsular bag dislocation and Nd: YAG anterior capsulotomy patients (Figure 5 and Figure 6).

## 4. Discussion

Studies of ACO after cataract surgery in non-pathological eyes typically report slight reductions in the opened area during the first post-operative months. In 78 patients receiving soft acrylic IOLs, the mean ACO reduction was 5% in the first month, 10% at the third month, and 8% at the sixth month [36]. Corydon et al. observed a similar reduction of 11% in AcrySof recipients in the 3rd month after surgery [37], while Sickenberg et al. reported a mean decrease of 3% in continuus curvilinear capsulorhexis (CCC) surface over 180 days after AcrySof implantation [38]. Park et al. reported 5%, 9%, and 10% reductions in ACO at one, three, and six months, respectively, when using one-piece acrylics [39]. All of these studies involved 4.5 to 5.5 diameter CCC. 

The average capsulorrhexis area for research patients was 14.3 mm^2^ during surgery, which is markedly smaller than the usual capsulorrhexis area created during an uncomplicated cataract operation, which is usually 5–5.5 mm [32,33,34,35] in diameter, corresponding to a 19.625–23.75 mm^2^ area. A small capsulorrhexis area for research patients can be explained by two factors—pronounced lens mobility due to weakened or non-existing zonules [28,31] and the surgical technique used in which the iris or capsular hooks (retractors) are attached to the capsulorrhexis (and consequently, the entire lens) edge. According to the author’s observations, a small capsulorrhexis provides a smaller attachment angle between the attaching hooks and the surface of the lens’ capsule, and therefore, there is less lens and anterior chamber mobility (fluctuations) during surgery, significantly reducing the risk of rupture to the anterior capsule. A small initial capsulorrhexis is an increased risk factor for contraction of the anterior capsule [40], which in combination with zonular weakness, can cause marked contraction of the anterior capsule with a deterioration or loss of vision, a change in the location of the IOL and the location angle and late IOL dislocation [3,12,34,41,42,43]. The approach used in the research when the small initial capsulorrhexis was maintained without enlargement, was based on the use of a CTR and a hydrophobic acrylic IOL with a sharp edge, a combination which reduces the risk of contraction of the anterior capsule [25,33,37,44,45]. In the research by Vasavada et al., in which 46 subluxated lens operations with a varying etiology of subluxation were analyzed, a surgical technique with a small initial capsulorrhexis was described, which had been enlarged to a standard size (5–5.5 mm in diameter) by the completion of surgery [28]. A standard initial capsulorrhexis size was reported in other subluxated lens research groups [26,46]. The capsulorrhexis of these study patients was not enlarged, as the author’s view is that this is an additional manipulation which can deepen an already weakened zonular defect or damage preserved zonules, aggravating the post-surgery outcome prognosis in this way. 

In contrast, anterior capsule opening contraction in high-risk eyes is more pronounced. Hayashi et al. studied cataract surgery patients with primary angle closure, PEX syndrome, or diabetic retinopathy, finding mean reductions from 8% to 16% in the first month after surgery, 18% to 29% in the 3rd month, and 17% to 30% at six months [34]. These results are in accordance with previous findings from the same authors, in cases of PEX syndrome, ACO contraction was 17.5% at 1st month and 23.9% at 6th month [47], and diabetic patients have also been noted to suffer statistically significant ACO reduction at the 3rd and 6th post-operative months [48]. 

Despite all of our patients having at least one risk factor for capsular contraction (e.g., weak zonules) [12], the 15% mean reduction in ACO during the six month follow-up was close to rates in non-pathological eyes (Figure 2). The use of capsular tension rings may be a factor, as Price et al. observed smaller rates (3%) of severe capsular contraction with the use of CTR [49] when compared to studies of patients at high risk for capsule contraction but without CTR implantation. For example, Hayashi observed marked ACO reduction in 13% of patients with PEX syndrome [47]. Further, in a retrospective study of patients with retinitis pigmentosa and cataract surgery, reduced capsular contraction syndrome was found in patients with CTR implantation compared to those without CTRs [24]. Shingleton et al., however, did not find a statistically significant difference in anterior capsular contraction after phacoemulsification and IOL implantation when comparing CTR implanted vs non-CTR implanted eyes with PEX related weak zonules [18]. Similarly, Takimoto et al. found only non-significant ACO reductions in eyes with implanted CTR compared to non-CTR controls [50]. 

Over the follow-up period, we found greater ACO reduction in patients with the 11 mm CTR (Figure 3). Takimoto et al. did not find significant differences in reduction between patients with 12.3 mm CTRs compared to those with 13 mm CTRs [50]. Similar levels of capsular bag reduction using 10 and 12 mm CTRs have been reported during the first follow-up month; after that period reductions continued only for the smaller CTRs [51]. However, the Strenn et al. study did not report ACO measurements or results for other types (e.g., silicone) of IOL. 

Seven of our patients (13%) suffered late IOL-CTR-capsular bag dislocation during the extended follow-up period (Table 3). Monastam reported a low rate (1%) of late IOL dislocation during a 20 year follow-up of 800 cataract patients with surgeries performed in 1997 and 1998 [6]. The timing of late IOL dislocation ranged from 3 to 19 years after the surgery. Eleven of the patients had received CTRs during their surgery, with two (18%) eventually experiencing late IOL-CTR-capsular bag dislocation. This percentage is higher than observed in our patients; however, Monastam’s study had a longer follow-up period and did not report the actual timing of the late dislocations [6]. Our patients experienced the first cases of late IOL dislocation within the first three years of follow-up [11], much earlier than reported for Monastam’s study (Table 3). We expect to find additional dislocations during our continuing follow-up.

A 2013 retrospective study from Lithuania found a mean time of 67 months from cataract surgery to late IOL dislocation [2]. This study included patients with PEX syndrome (57%), intraoperative zonular weakness (35%), CTR implantation (29%), and trauma (22%). Gunenc et al. reported two cases of late IOL-CTR-capsular bag dislocations six years after the initial cataract surgery. The article also reported a simple technique for IOL-CTR-capsular bag repositioning by suturing it to the sulcus in twelve o’clock position at the edge of the CTR using 10/0 polypropylene suture [52]; we used the same approach [11]. 

Three case reports by Kocak et al. present late IOL-CTR-capsular bag dislocation at 2.5, 3, and 8 years after cataract surgery for patients with PEX syndrome-associated weak zonules. All three patients had received hydrophilic acrylic lenses and developed marked fibrosis in the anterior capsule opening and capsular bag [10]. Shingleton et al. (2017) reported three cases (2%) of late IOL dislocation among 143 high-risk patients (PEX syndrome with pre- and intra-operative diagnosed zonular weakness) but did not provide the timing of the dislocation or whether CTRs had been implanted. In the 76 patient control group (PEX syndrome, no signs of zonular weakness), one patient (1%) suffered a late IOL dislocation [18]. The same study reported that there were no statistical differences in the occurrence of late complications, including late IOL dislocation, between high risk patients with or without CTR implantation [18]. 

In our study, one case of late IOL-CTR-capsular bag dislocation due to rupture of the polypropylene suture occurred 24 months (two years) after surgery with an implanted Cionni CTR (Table 3). The timing of the rupture coincides with the mean rupture time after surgery (18 months) reported by Cionni et al. in 90 eyes (57 patients) with congenital subluxated lenses [53]. Moreover, 10% (nine eyes) experienced late IOL-CTR-capsular bag dislocation, very close to our results. Cheung et al. reported on a patient with posttraumatic lens subluxation, Cionni ring implantation, and a 9/0 polypropylene suture with late IOL-CTR-capsular bag dislocation due to suture rupture six years after surgery [54]. The authors also reported that resuturing with the same 9/0 polypropylene material is possible.

Capsular bag contraction and ACO reduction may be among the causes of late IOL-CTR-capsular bag dislocation [4]. Our patients with late IOL-CTR-capsular bag dislocation experienced a mean ACO reduction of 33% during the first six months (Figure 5). Monestam did not perform ACO measurements during or after the cataract surgeries, but did not observe capsular contraction syndrome requiring Nd: YAG anterior capsulotomy in the 800 patient cohort, including those with late IOL-capsular bag dislocation [6]. Davis et al. reported capsular contraction syndrome and phimosis of capsulorhexis as frequent findings in patients with late IOL dislocation [1]. The conditions were detected in 18 of 43 specimens (42%) with PEX syndrome and 5 of 16 specimens (31%) after vitrectomy, however, CTRs had not been implanted in the initial cataract surgery. Marked phimosis of ACO in cases of late IOL-CTR-capsular bag dislocation was also reported by Kocak [10]. In their group of 76 patients with preoperative signs of PEX-associated zonular weakness, similar to our patient group, significant phimosis requiring Nd: YAG anterior laser capsulotomy occurred in six eyes (7.6%) and late IOL dislocation was reported in an additional eye (1.3%) [18]. Other studies have reported PEX syndrome as a major risk factor for late IOL-capsular bag dislocation [2,3,9,54]. Capsular and anterior capsule openings were not analyzed in these studies, but capsular contraction and phimosis occlusion of the capsulorhexis opening after cataract surgery is a well-known characteristic of PEX syndrome [18,34,55].

Eight of our patients (15%) received Nd: YAG anterior capsulotomies, almost all during the first 12 months after the initial surgery (Figure 4) and with a mean ACO reduction of 33% during the first six months (Figure 6). Hayashi et al. observed a marked ACO reduction in PEX syndrome patients—9% experienced decreases in the ACO opening to below 10 mm^2^ during the first 12 months after surgery and an Nd: YAG anterior capsulotomy [47]. Ye et al. analyzed 11 consecutive cases of anterior capsular opening phimosis after cataract surgery. In that study, Nd: YAG anterior capsulotomies were performed an average of four months after surgery and the ACO opening diameter prior to laser treatment was 2.2 mm. Progressive zonular insufficiency was observed in two patients but no late IOL-capsular bags dislocations occurred during the study period [56]. Among the 11 patients, nine (82%) had a major risk factor (diabetes mellitus, high myopia, or retinitis pigmentosa) for anterior capsular contraction [24,34,57,58]. In our study, two patients (N21 and N26) with early capsular phimosis and Nd: YAG anterior capsulotomy at two months and one month post-surgery, respectively, experienced late IOL-CTR-capsular bag dislocation at three and seven years, respectively (Table 3). This may implicate early and high ACO reduction rates as additional risk factors for late IOL dislocation. 

The study limitations include a relatively small number of participants, short follow-up time (5–9 years) for late complications, and absence of surgeon himself in observations of a number of the studied patients, potentially leading to a loss of additional significant data. 

The study strength is that we have demonstrated/identified/outlined additional data for late IOL dislocations and their connection to ACO contraction in a group of cataract surgery patients with weak or partially absent zonular support.

## 5. Conclusions

In conclusion, conducting successful cataract surgery in cases of weak or absent lens zonules is challenging but possible with the use of additional capsular bag stabilization devices (hooks and CTRs). The use of hydrophobic acrylic lenses and CTR reduces, but does not eliminate, anterior capsule contraction. Contraction rates after cataract surgery are comparable to those in patients without ocular comorbidity, even in cases of small initial capsular opening sizes and additional non-washing of LEC. Although our study patients experienced a relatively high rate of late IOL-CTR-capsular bag dislocation, phacoemulsification with CTR implantation is still justified as the majority of patients did not develop complications. Additionally, dislocated complexes are easily repositioned and few patients will require IOL exchange with or without additional vitrectomy. Frequent long-term follow-up visits should be considered to deal with potential late complications in such patients. 

## Figures and Tables

**Table 1:** Patients’ baseline characteristics.

	Overall (*N* = 53)	*p* Value *
**Sex**		
Male	41 (77.4%)	<0.001
Female	12 (22.6%)	
**Implicated eye**		
Right eye	22 (41.5%)	0.27 (n.s.)
Left eye	31 (58.5%)	
**Glaucoma**		
No	30 (56.6%)	0.41 (n.s.)
Yes	23 (43.4%)	
**Myopia**		
No	47 (88.7%)	<0.001
Yes	6 (11.3%)	
**History of trauma**		
No	36 (67.9%)	0.01
Yes	17 (32.1%)	
**Marfan syndrome**		
No	50 (94.3%)	<0.001
Yes	3 (5.7%)	
**PEX syndrome**		
No	28 (52.8%)	0.78 (n.s.)
Yes	25 (47.2%)	

* Significance level of results based on binominal test.

**Table 2:** Area of anterior capsule opening (ACO) at post-operative follow-up visits.

	Mean (SD)	*p* Value
1 day	14.3 (3.68)	
1 week	14.2 (3.48)	
1 month	13.6 (3.80)	<0.001
3 months	12.5 (4.45)	
6 months	12.1 (3.68)	

**Table 3:** Demographic and clinical data of seven patients with in–the–bag intraocular lens dislocation.

Case No. (Study No)	Age during Primary Surgery (Yrs)	Gender	Time from Surgery to Dislocation (Yrs)	Predisposing Conditions (Diagnosis/Associated Presentation)	Type of CTR Implanted During Primary Surgery	Additional Manipulations	Outcome
1 (N7)	79	Male	7	Blunt trauma	CTR		IOL-CTR-capsular bag removal, open loop IOL implantation in anterior chamber
2 (N9)	48	Female	2	Marfan syndrome	Modified CTR (Cionni), 2 fixation arms		Removal of IOL-CTR-capsular bag complex, aphakic
3 (N21)	80	Female	3	PEX	CTR	ACO contraction 2 months after initial surgery, Nd: YAG laser capsulotomy at this point	IOL-CTR-capsular bag reposition, endophthalmitis after reposition
4 (N25)	71	Female	5		CTR	Posterior capsule o pacification (PCO) Nd: YAG posterior laser capsulotomy	IOL-CTR-capsular bag dropped in vitreous during exchange or reposition attempt, other clinic
5 (N26)	74	Male	7	PEX, glaucoma	CTR	ACO contraction 1 months after initial surgery, Nd: YAG laser anterior capsulotomy at this point	IOL-CTR-capsular bag exchange, open loop AC IOL implantation, other clinic
6 (N40)	49	Female	8	Blunt trauma	CTR	PCO Nd: YAG posterior laser capsulotomy 3 months after surgery	IOL-CTR-capsular bag complex reposition
7 (N50)	77	Male	1	Blunt trauma, PEX	CTR		IOL-CTR-capsular bag complex reposition

CTR: capsular tension ring, IOL: Late intraocular lens, PEX: pseudoexfoliation, ACO : anterior capsule opening.

## Data Availability

The data that support the findings of this study are available from the corresponding author under reasonable request.
